# Preservation of sexual and reproductive function in the treatment of extragonadal yolk sac tumors in the female genital tract

**DOI:** 10.3389/fped.2022.1004501

**Published:** 2022-11-14

**Authors:** Xiao Ma, Dongyan Cao, Peng Peng, Yu Xiao, Jiaxin Yang, Huifang Huang, Ying Zhang, Mei Yu, Jinhui Wang, Huimei Zhou, Ninghai Cheng, Tao Wang, Keng Shen

**Affiliations:** ^1^ Department of Obstetrics and Gynecology, National Clinical Research Center for Obstetric & Gynecologic Diseases, Peking Union Medical College Hospital, Chinese Academy of Medical Sciences & Peking Union Medical College, Beijing, China; ^2^ Department of Pathology, Peking Union Medical College Hospital, Chinese Academy of Medical Science and Peking Union Medical College, Beijing, China

**Keywords:** extragonadal, germ cell tumors (GCTs), paediatric [MeSH], fertility preservation, menstruation, yolk sac tumor (YST)

## Abstract

**Objective:**

This study aimed to summarize the clinical features, treatment modalities, therapeutic effects, menstruation and fertility outcomes, and prognosis of extragonadal yolk sac tumors (YSTs) of the female genital tract.

**Methods:**

We reviewed 32 cases of extragonadal YSTs in the genital tract treated between 1983 and 2021. The medical records, including clinical characteristics, histopathology, treatments, chemo-reduced adverse events, and outcomes on long-term follow-up, were collected.

**Results:**

Among the 32 cases, 30 were vaginal YSTs and two were uterine YSTs (endometrial and cervical). Thirty patients (30/32, 93.8%) were <4 years. Abnormal vaginal bleeding (*n* = 31) and elevated serum alpha-fetoprotein level (*n* = 32) were the most common presentations. Vaginohysteroscopy and/or pediatric rhinoscopy were used for diagnosis in 17 pediatric patients and evaluation of chemotherapeutic efficacy in 21 pediatric patients. All the patients received combination chemotherapy. Bleomycin/etoposide/cisplatin (BEP) was chosen with prior consideration in 28 cases; 21 patients were treated with BEP alone. Yellow or grayish-yellow tissue with irregular shape was found in 66.7% of the cases during repeat examinations. Five patients underwent surgeries during repeat examinations and follow-ups, and no evidence of malignancy was noted in them. Thirty-one patients achieved complete remission. During a median follow-up of 63 months (2.4–240.3 months), two patients experienced recurrence, three died, and 29 remained disease-free. One patient recovered menstruation and five had undergone menarche.

**Conclusion:**

BEP chemotherapy can serve as a preferred treatment modality for vaginal and uterine YSTs. Vaginohysteroscopy and pediatric rhinoscopy can be used for diagnosis and evaluation of chemotherapeutic efficacy in pediatric patients. YSTs possibly appear as yellow or grayish-yellow after chemotherapy.

## Introduction

Yolk sac tumors (YSTs) are usually encountered in ovaries and account for approximately 1% of all ovarian malignancies (1, 2). Less commonly, YST can arise from extragonadal sites, particularly in the midline of the body (3). Within the female genital tract, the vagina is the most common site of extragonadal YSTs, followed by the uterus and vulva (4, 5). A total of 137 cases of vaginal YSTs have been reported over a 100-year period (6). YSTs of the uterus are even more scarce, and fewer than 40 endometrial YST cases have been reported (7–28). Unexplained vaginal bleeding and necrotic masses protruding from the vagina are the main clinical manifestations of vaginal and uterine YSTs (18).

Vaginal YSTs are locally aggressive and capable of hematogenous and lymphatic metastasis (22, 23, 29, 30). Early detection and appropriate therapy are key to improving prognosis. Historically, vaginal YSTs have been treated with radical surgery and irradiation, which leads to a loss of reproductive function and results in a poor prognosis. With the introduction of the bleomycin/etoposide/cisplatin (BEP) regimen in the 1980s, treatment has shifted toward chemotherapy with or without conservative surgery (6, 31, 32), leading to a favorable prognosis (18) without sacrificing fertility or sexual function. Due to the rarity of endometrial and cervical YSTs, the reported cases were treated with various types of surgery, with or without adjuvant therapy. Chemotherapy regimens varied from case to case.

Chemotherapy, especially BEP chemotherapy, is the only treatment modality that preserves sexual and reproductive function in vaginal YSTs (30, 33). However, long-term outcomes, including data on menstruation and fertility after vaginal YSTs treatment, are not available. Additionally, there is a paucity of literature summarizing the clinical experience of diagnosis, management, and evaluation in pediatric patients. Furthermore, standard treatments for endometrial and cervical YSTs have not yet been established. Whether radical surgery is mandatory and whether fertility-sparing treatment is suitable for uterine YSTs requires further investigation.

Herein, we share our experience with the diagnosis and treatment of extragonadal YSTs originating from the female genital tract and show the long-term outcomes of menstruation and fertility.

## Materials and methods

### Ethical approval

This study was approved by the Institutional Review Board of Peking Union Medical College Hospital (approval number: S-K2102). Since the study was retrospective and fully anonymous, the requirement for written informed consent was waived.

### Patients

We searched the electronic database and reviewed female patients with primary extragonadal malignant germ cell tumors (EMGCTs) who were treated in our hospital between 1983 and 2021. Patients with primary EMGCTs were identified during this period, in whom patients with EMGCTs outside the female genital tract were excluded, and patients with EMGCT in the female genital tract were included in the present study.

### Clinicopathologic data

Clinical and demographic data, including age at onset, presenting complaints, clinical features, radiologic findings, tumor markers, pathology, treatment modality, and prognosis, were collected from the medical records and *via* telephone. Histological slides were reviewed by two independent gynecological pathologists from the Department of Pathology at our hospital. Information on survival outcome, menstruation, and fertility was collected on March 23, 2022, using both medical records and telephone follow-up.

### Outcomes and follow-up

Complete remission (CR) was defined by either a normal serum alpha-fetoprotein level (defined in our hospital <20 U/ml) or a negative pathology result. The patients achieving complete remission were under close follow-up at the outpatient department. A routine medical examination, including physical examination, serum alpha-fetoprotein level monitoring, and pelvic and abdominal ultrasound, was performed at each follow-up visit. The patients were followed up according to the following plan: monthly during the first year, every 2 months during the second year, every 3 months during the third and fourth years, and twice a year during the fifth year and thereafter.

### Statistics

Descriptive statistics was used to describe the patients’ characteristics. Continuous variables are presented as mean ± SD or median (range) and categorical data are presented as number (percentage). Statistical analysis was performed using SPSS 25.0 statistical software (SPSS Inc., Chicago, IL, USA).

## Results

### Clinical features

A total of 209 patients with primary extragonadal malignant germ cell tumors were identified, 177 patients with such tumors outside the female genital tract were excluded and 32 (15.3%) with tumors in the female genital tract were included. The clinical features of these 32 patients are presented in Table 1. All of 32 patients were histopathologically diagnosed with YST. Except for one 29-year-old patient with a vaginal YST, the remaining 29 patients with vaginal YSTs were all under 4 years. The patient ages at diagnosis of endometrial and cervical YST were 2 and 31 years, respectively. Among 31 (31/32, 96.88%) patients with a complaint of AVB or mucosanguineous discharge, 11 patients had necrotic tumor tissue protruding from the vagina, and one patient had difficulty passing urine concurrently. Preoperative serum alpha-fetoprotein (AFP) levels were markedly elevated in all patients (range, 251–54,000 ng/ml). Except for one patient whose imaging studies were unavailable, the other 31 patients underwent at least one type of pelvic imaging on initial presentation, including pelvic ultrasound, computed tomography, magnetic resonance imaging, and/or positron emission tomography–computed tomography. Twenty patients (64.5%) underwent general imaging evaluation (chest-abdomen-pelvis). Only two patients had radiologic metastases at diagnosis: one patient had minor nodules in the lower lobe of the lungs on x-ray, and another patient had multiple tumor nodules located on the peritoneal surface on computed tomography.

**Table 1:** The clinical features of 32 patients in the present study.

Age (*n* = 32)	10 months (3 months to 32 year)
Prepubertal and nulliparous (*n* = 30)	
≤3 years	29 (90.6%)
3.5 years	1 (3.1%)
Adults (*n* = 2)	
>18 years	2 (6.25%)
Chief complaint (*n* = 32)	
Abnormal vaginal bleeding or mucosanguineous discharge	31 (96.9%)
Protuding masses from the vagina	11 (34.4%)
Contact bleeding	1 (3.1%)
Urination defecation difficulties	1 (3.1%)
AFP, ng/ml (*n* = 32)	3965.5 (251–54,000)
Tumor site (*n* = 32)	
Vagina	30 (93.8%)
Endometrium	1 (3.1%)
Cervix	1 (3.1%)
Tumor size, cm (*n* = 32)	4.5 (1–9)
Extent of diseases (*n* = 32)	
Localized	30 (93.8%)
Metastatic^a^	2 (6.3%)
Fertility-sparing	
Yes	30 (93.8%)
No	2 (6.3%)
Chemotherapy (*n* = 32)	
BEP alone	21 (65.6%)
BEP→Other regimens	7 (21.9%)
Other regimens→BEP or other	4 (12.5%)
Response to initial treatment (*n* = 32)	
CR	31 (96.9%)
Uncontrolled	1 (3.1%)
Follow-up, months	63 (2.4–240.3)
Death rate	3 (9.4%)

BEP, bleomycin/etoposide/cisplatin; AFP, alpha-fetoprotein; CR, complete remission.

^a^ Radiologic metastases.

### Diagnosis and histological findings

**Figure 1:**
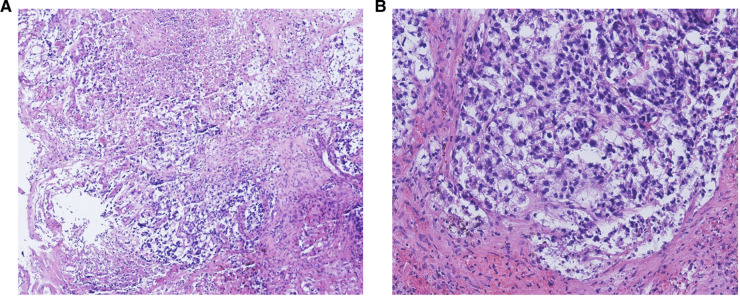
The morphologic features of yolk sac tumors (YSTs). (A) A large necrotic area is shown. Hematoxylin and eosin stain. Magnification, 100×. (B) A reticular pattern, with clear cytoplasm and less nuclear atypia, is shown. Hematoxylin and eosin stain. Magnification, 100×.

**Figure 2:**
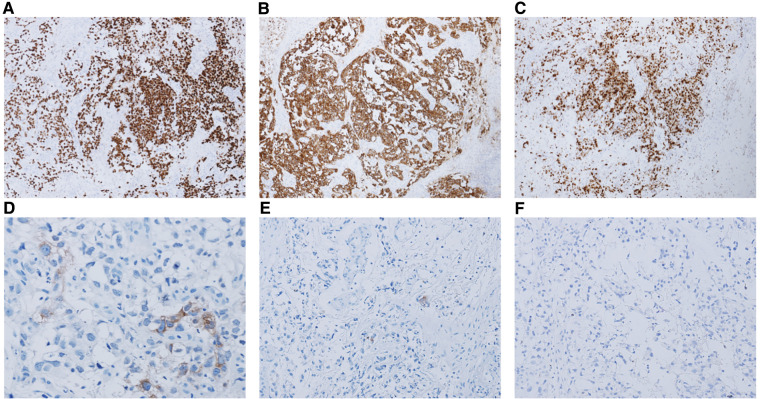
The immunohistochemical results of some cases that were tested. (A) Immunohistochemistry (IHC) for SALL-4 showing positive staining. Magnification, 100×. (B) IHC for CK AE1/AE3 + showing positive staining. Magnification, 100×. (C) Ki-67 index is approximately 90%. Magnification, 100×. (D) IHC for AFP showing focally positive staining. Magnification, 100×. (E) IHC for OCT 3/4 showing almost negative staining. Magnification, 100×. (F) IHC for CD30 showing negative staining. Magnification, 100×.

Tissue biopsy was performed in all 32 cases, and all cases were pure YST (100%). Figure 1 shows the morphological features of YST in one case. Pathological specimens were obtained from exfoliated tissues in 10 patients (31.3%), tumor biopsy in 19 patients (59.4%), and tumor resection in 3 patients (9.7%). Because the anatomical structures of pediatric patients are generally small, the vaginoscopic approach for hysteroscopy and pediatric rhinoscopy were used to inspect and obtain biopsies through the magnification of endoscopic images. Regarding tumor biopsy, an ultrasound-guided biopsy of the pelvic mass was performed in the case of endometrial YST; multiple cervical biopsies were performed by colposcopy in the case of cervical YST; the biopsy specimens of vaginal YSTs were obtained by vaginohysteroscopy in nine cases, biopsy forceps in five cases, pediatric rhinoscopy in two cases, and a combination of vaginohysteroscopy and pediatric rhinoscopy in one case (as shown in Table 2 and Figure 2). Immunohistochemical staining served as a helpful adjunctive tool for the diagnosis of YST in 22 of the 32 cases. Table 2 and Figure 2 shows the immunohistochemical results of the tested cases.

**Table 2:** The diagnostic information and immunohistochemical results.

Histological subtype (*n* = 32)	
Pure YST	32 (100%)
Pathologic diagnosis (*n* = 32)	
Before chemotherapy	30 (93.8%)
After chemotherapy	2 (6.2%)
Specimen origination (*n* = 32)	
Biopsy	19 (59.4%)
Exfoliated tissues	10 (31.3%)
Laparatomy tumor recection	3 (9.7%)
Means of biopsy (*n* = 19)	
Vaginohysteroscopy	9 (47.4%)
Biopsy forceps	5 (26.3%)
Pediatric rhinoscopy	2 (10.5%)
Biopsy under ultrasonographic guidance	1 (5.3%)
Colposcopy	1 (5.3%)
Vaginohysteroscopy + Pediatric rhinoscopy	1 (5.3%)
Immunohistochemical results (*n* = 22)	
The positive rate, %	
CK AE1/AE3	100% (14/14)
SALL4	100% (7/7)
AFP	94.7% (18/19)
Glypican-3	80% (4/5)
PLAP	75% (6/8)
CD117	55.6% (5/9)
The negative rate, %	
CD30	100% (13/13)
OCT3/4	100% (11/11)
CK7	100% (5/5)
MYOD1	100% (3/3)
D2-40	100% (3/3)
EMA	87.5% (7/8)
HCG	85.7% (6/7)
SOX2	75% (6/7)
Ki-67, %	61.25 (2–98)

YST, yolk sac tumor; CK AE1/AE3, cytokeratin; SALL4, spalt like transcription factor 4; AFP, alpha-fetoprotein; PLAP, placental-like alkaline phosphatase; OCT 3/4, octamer binding transcription factor; CK7, cytokeratin 7; MYOD1, myogenic differentiation 1; EMA, epithelial Membrane Antibody; HCG, human chorionic gonadotropin.

### Treatment

Table 3 shows detailed therapeutic information for each case. Nine patients (28.1%) were initially admitted to local hospitals and were subsequently referred to our hospital. Twenty-three patients (71.9%) were exclusively diagnosed with and treated in our hospital. Two patients with vaginal YSTs underwent surgery at local hospitals prior to chemotherapy. One patient (No. 24) underwent radical surgery, including vaginal tumorectomy, pelvic lymphadenectomy, and internal iliac venous embolectomy, and was administered a cycle of nedaplatin, vindesine, etoposide, and bleomycin (NVEB) postoperatively. Another patient (No. 18) underwent vaginal tumorectomy *via* laparotomy, but she did not receive any postoperative chemotherapy in the local hospital.

**Table 3:** Detailed therapeutic and follow-up information for each case.

No.	Venous infusion ports	Treatment modality	Chemotherapy regimen in local hospitals	Chemotherapy regimen in our hospital	Total cycles	Cycles before AFP normalized	Cycles after AFP normalized	Status	Outcome	Follow-up time (months)
1^a^	Yes	Chemo*3→Evaluation→Chemo	BEP*3	BEP*5	8	4	4	CR	Alive	50.4
2	No	Chemo*3→Evaluation→Chemo	/	BEP*4	4	2	2	CR	Alive	30.3
3	Yes	Chemo*3→Evaluation→Chemo	/	BEP*5	5	3	2	CR	Relapse in vagina, CR and alive	75.8
4	Yes	Chemo*7→Evaluation→Chemo	/	BEP*7→PVB*1	8	4	4	CR	Alive	45.5
5	Yes	Chemo*4→Evaluation→Chemo	/	BEP*5	5	3	2	CR	Alive	55.8
6^a^	Yes	Biopsy several times combined with chemotherapy	JEB*6→PEV*2→BEP*1	BEP*4	13	3	/	Uncontrolled	Dead	27.9
7^a^	Yes	Chemo*2→Evaluation	BEP*2	/	2	2	0	CR	Alive	2.4
8	Yes	Chemo*4→Evaluation→Chemo	/	BEP*4→TIP*4	8	8	0	CR	Alive	33.6
9	Yes	Chemo*6	/	BEP*5→PE*1	6	4	2	CR	Alive	89.1
10	No	Chemo*2→Evaluation→Chemo	/	BEP*4	4	2	2	CR	Alive	53.7
11	Yes	Chemo*4	/	BEP*4	4	3	1	CR	Dead	3.6
12	Yes	Chemo*4→Evaluation	/	BEP*4	4	2	2	CR	Alive	60.4
13^a^	Yes	Chemo*1→Evaluation→Chemo	BEP*1	BEP*3	4	2	2	CR	Alive	30.1
14	No	Chemo*4→Evaluation→Chemo	/	BEP*5→PEV*1→VAC*1	7	2	5	CR	Alive	236.0
15	No	Chemo*3→Evaluation→Chemo	/	BEP*5	5	3	2	CR	Alive	164.3
16	Yes	Chemo*3→Evaluation→Chemo	/	BEP*6	6	3	3	CR	Alive	143.5
17	Yes	Chemo*4	/	BEP*4	4	2	2	CR	Alive	74.1
18^a^	Yes	Chemo*3→Evaluation→Chemo	/	BEP*5	5	2	3	CR	Alive	65.9
19	Yes	Chemo*4→Evaluation→Chemo	/	BEP*1→PEV*2→BEP*1→VAC*1	5	2	3	CR	Alive	116.7
20	Yes	Chemo*3→Evaluation→Chemo	/	BEP*5	5	3	2	CR	Alive	114.4
21	Yes	Chemo*2→Evaluation→Chemo	/	BEP*4	4	3	1	CR	Alive	100.6
22	Yes	Chemo*2→Evaluation→Chemo	/	BEP*4	4	2	2	CR	Alive	91.5
23	Yes	Chemo*3→Evaluation→Chemo	/	BEP*4	4	2	2	CR	Alive	92.2
24^a^	Yes	Chemo*3	NVEB*1	BEP*2	3	1	2	CR	Alive	84.9
25^a^	Yes	Chemo*3→Evaluation→Chemo	VBEP*2→JEB*1	BEP*2	5	3	2	CR	Alive	67.6
26	Yes	Chemo*2→Evaluation→Chemo	/	BEP*4→TIP*2	6	4	2	CR	Alive	40.5
27	No	Chemo*2→Evaluation→Chemo	/	BEP*4	4	2	2	CR	Alive	32.1
28	No	Chemo*7→Evaluation→Chemo	/	PVB*3→PEV*5→VAC*1	9	3	6	CR	Alive	240.3
29^a^	No	Chemo*6→Evaluation→Chemo	BEP*10	/	10	3	7	CR	Alive	33.4
30^a^	No	Chemo*4→Evaluation→Chemo	BEP*4	BEP*2	6	3	3	CR	Alive	25.2
31	No	Chemo*3→Evaluation→Chemo	/	BEP*4→PEV*1	5	3	2	CR	LHBS after completing completion, no evidence of YST; Alive	67.2
32	Yes	Chemo*5	/	BEP *5	5	3	2	CR	Relapse twice in vagina, Dead finally.	23.4

Chemo, chemotherapy; AFP, alpha-fetoprotein; BEP, bleomycin/etoposide/cisplatin; PVB, cisplatin/vincristine/bleomycin; PE, cisplatin/epirubicin; PEV, cisplatin/etoposide/vincristine; TIP, paclitaxel/ifosfamide/cisplatin; VAC, vincristine/actinomycin/cyclophosphamide; JEB, carboplatin/etoposide/bleomycin; NVEB, nedaplatin/vindesine/etoposide/bleomycin; VPEB, vincristine/cisplatin/etoposide/bleomycin; CR, complete remission; LHBS, laparoscopic hysterectomy and bilateral salpingectomy.

^a^ Patients who were transferred from other hospitals.

All 32 patients underwent combination chemotherapy: 30 patients started chemotherapy following pathologic diagnoses, and two patients received chemotherapy after clinical diagnoses based on high AFP levels and imaging findings. Excluding two patients who underwent surgery in local hospitals, the remaining 30 patients initially underwent chemotherapy alone. With regard to chemotherapy regimens, 28 of the 32 patients (87.5%) were initially started on a regimen of BEP (bleomycin/etoposide/cisplatin) chemotherapy. Among them, 21 patients were treated with BEP alone; 7 patients subsequently changed from BEP to other regimens, including PVB (cisplatin/vincristine/bleomycin)/TIP (paclitaxel/ifosfamide/cisplatin)/PE (cisplatin/epirubicin)/PEV (cisplatin/etoposide/vincristine)/VAC (vincristine/actinomycin/cyclophosphamide). Usually, the chemotherapy protocol was changed from BEP to other regimens when patients reached the individualized lifetime maximal dose of bleomycin (250 mg/m^2^) or cannot tolerate bleomycin-related pulmonary toxicity (Table 3). Four of the 32 patients (12.5%) were started on a non-BEP regimen, including NVEB, JEB (carboplatin/etoposide/bleomycin), VPEB (vincristine/cisplatin/etoposide/bleomycin), and PVB. Three patients were transferred from local hospitals to our hospital. After routine evaluation in our hospital, chemotherapy was continued in these three cases, in which the BEP regimen was chosen with prior consideration, since the bleomycin dosage was within the individualized lifetime maximal dose (250 mg/m^2^). The patient who was started on a PVB regimen was exclusively treated in our hospital. Her chemotherapy protocol was subsequently changed from PVB to both PEV and VAC to prevent potential bleomycin lung toxicity.

Due to our inexperience with pediatric nursing, we worked in close collaboration with pediatricians and nurses in the management of children. To provide nutritional support and ensure the efficacy and safety of intravenous (IV) chemotherapy, IV infusion ports were implanted subcutaneously in 79.3% of the pediatric patients. In addition to providing nutritional support, IV infusion ports can prevent repeated vascular punctures, reduce the risks of drug extravasation, and protect blood vessels. To prevent cisplatin nephrotoxicity during chemotherapy, 1/3–1/4 tonic fluid (60–80 ml/kg, no more than 100 ml/kg) was administered intravenously at 10–15 drip/min.

### Evaluation of treatment efficacy

In the current study, elevated preoperative serum AFP levels were found in all 32 patients. As a sensitive tumor marker for YSTs, serum AFP was measured every course to evaluate tumor response. Chemotherapy was considered satisfactory if the serum AFP level decreased by 10^1^ every course. The normal serum AFP level was <20 U/ml. The median number of total cycles of chemotherapy was five cycles (range, 2–13 cycles), the median number before AFP dropped to normal values was three cycles (range, 1–8 cycles), and the median number after a normalized AFP was two cycles (range, 0–7 cycles). Repeat examinations under anesthesia were usually performed to evaluate tumor response after AFP levels returned to normal levels. A delayed decrease in AFP levels and any suspicious radiological findings were also reasons for repeat evaluation.

**Figure 3:**
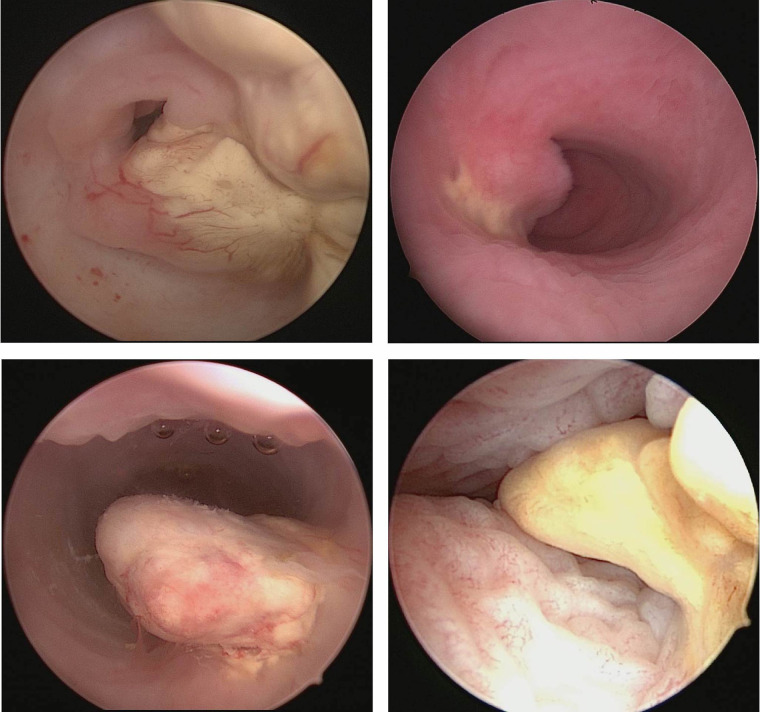
Yellow, grayish-yellow, or yellowish-white tissue varying in size and shape.

A total of 27 patients underwent repeat examinations under anesthesia, of whom 22 had normalized serum AFP levels and 5 had abnormal or near-normal levels. Vaginohysteroscopy was used in 20 cases, and pediatric rhinoscopy and laparoscopy were used separately in 4 cases, either alone or in combination with vaginohysteroscopy. Yellow, grayish-yellow, or yellowish-white tissue varying in size and shape was the most common intraoperative finding during repeat examinations and was found in 18 cases (66.7%) (Figure 3). During repeat examinations under anesthesia, 23 patients underwent biopsies and 4 patients underwent additional procedures due to highly suspected residual disease, including partial vaginectomy (*n* = 2), vaginal tumorectomy (*n* = 1), and total hysterectomy plus bilateral salpingectomy (THBS) (*n* = 1). With regard to the pathological results, no evidence of malignancy was found in 25 cases, a little necrotic and degenerated tumor tissue was found in one case, and persistent disease was confirmed in only one uncontrolled case.

### Adverse effects of chemotherapy

In the current study, myelosuppression, chemotherapy-induced nausea, and vomiting were the most common side effects. Oral ulceration, anorexia, hair loss, and bleomycin-induced interstitial disease were the least common side effects observed. To improve efficacy and reduce chemotherapy-induced adverse effects, supportive care was provided to patients, such as blood transfusion, platelet transfusion, anti-inflammatory treatment, and granulocyte colony-stimulating factor (G-CSF) support. For pediatric patients, G-CSF was administered when the neutrophil count dropped below 0.5 × 10^9^/L with a dosage of 2 μg/kg. The volume of blood transfused for children was 10–15 ml/kg. The vast majority of patients tolerated the toxic effects of multidrug chemotherapy well, except for one patient (No. 11). Her serum AFP level decreased to a normal value after three cycles of BEP, but she developed a fourth degree of bone marrow suppression, severe nausea and vomiting, and pulmonary function decline. Unfortunately, she progressed to acute dyspnea and heart failure after the fourth cycle of BEP (her cumulative dose of bleomycin was 44 mg) and finally died of respiratory and circulatory failure.

### Outcomes and follow-up

During a median follow-up of 63 months (2.4 months to 19.8 years), four (12.5%) patients showed disease progression. Details of the patients with disease progression are shown in Table 4. Thirty-one (96.88%) patients achieved CR after the initial treatment, but one of them (No. 11) died of chemo-related severe adverse events. One patient (No. 6) had uncontrolled disease after the initial treatment and died of the disease. Two patients (No. 3 and 32) experienced recurrence, and one of them (No. 32) died of recurrent disease. The patient with a cervical YST underwent laparoscopic THBS because of radiologic metastasis during follow-up. However, the final pathological diagnosis revealed no evidence of malignancy. To date, three patients (3/32, 9.38%) died, and the remaining 29 patients (29/32, 90.63%) are alive without evidence of disease. In general, we achieved an excellent prognosis (Table 1).

**Table 4:** Details of the patients with disease progression.

No.	Age	Primary tumor site	Extent of disease	Initial treatment	Time to relapse	Site of relapse	Treatment after relapse	Outcome
3	11 months	Vagina	Localized	Our hospital: EUA + hys TSB→BEP*3→EUA + hys TSB →BEP*2	3.5 months after completion of initial treatment	Vagina	BEP*5	NED (6.2 years)
6	29 months	Vagina	Localized	Local hospital: EUA + hys TSB→JEB*2→hys TSB→JEB*1→laparotomy exploration + TR→JEB*3→PEV*2→TSB pv→BEP*1→transferred to our hospital Our hospital: BEP*3→lap + hys + TR + LND→BEP*1	/	/	/	DOD (28 months)
11	9 months	Vagina	Peritoneal metastasis on imaging studies)	Our hospital: Diagnosed by exfoliated tissues→BEP*4 (DDP, 100 mg/m^2^/3 days; IV drip days 1–3 + VP-16, 100 mg/m^2^/day; IV drip days 1–3 + BLM, 15 mg/m^2^/day; intramuscular, day 1–2)	/	/	/	DOD (4 months)
32	9 months	Vagina	Localized	Our hospital: EUA + hys TSB→BEP*5	① The 1st relapse: 1.5 months after completion of initial treatment. ② The 2nd relapse: after VAC consolidation chemotherapy of the second-round chemotherapy	Vagina	① Hys TSB→BEP*4 + VAC*1 ② lap + TR→EMA/CO*1 + PVB*2 + PEV*4	DOD (23 months)

EUA, examination under anesthesia; hys, hysteroscopy; pv, per vagina; lap, laparoscopy; TSB, tumor site biopsy; TR, tumor resection; BEP, bleomycin/etoposide/cisplatin; JEB, carboplatin/etoposide/bleomycin; PEV, cisplatin/etoposide/vincristine; DDP, cisplatin; BLM, bleomycin; VAC, vincristine/actinomycin/cyclophosphamide; PVB, cisplatin/vincristine/bleomycin; EMA/CO, etoposide/methotrexate/actinomycinD/cyclophosphamide and vincristine; LND, lymphadenectomy; IV, intravenous; NED, no evidence of disease; DOD, died of disease.

In a total of 27 patients, including one childbearing patient and 26 children, the uterus and both ovaries were preserved. For these 27 patients, the median follow-up period was 67.6 months (2.4 months to 19.8 years). The childbearing patient recovered her menstruation three and a half months after completion of chemotherapy. Five of the 26 patients underwent menarche during the long-term follow-up. None of the 27 patients had conceived yet because of the lack of childbearing requirements.

## Discussion

Extragonadal YSTs of the lower female genital tract, as a small portion of EMGCTs, are even scarcer. The vagina is the most common site of extragonadal YSTs (4, 5, 34), which occurs exclusively in girls under 3 years of age (18). Vaginal YSTs are locally aggressive, and untreated patients could die within 2–4 months after the initial presentation (35); hence, timely initiation of optimal treatment plays a key role in improving the prognosis of vaginal YSTs. As the treatment of vaginal YSTs has shifted from radical surgery to chemotherapy, especially BEP chemotherapy, the diagnosis of vaginal YSTs has relied heavily on the protruding masses from the vagina or a biopsy specimen.

For patients presenting with protruding masses from the vagina, any exfoliated tissue should be taken for histological examination. Usually, lesions of the lower genital tract are diagnosed through speculum examination, during which careful inspection of the lower genital tract is performed, and a biopsy is obtained when indicated. Since vaginal YSTs mostly occur in children under 3 years, whose anatomical structures are generally small, the usual approach is not suitable even under anesthesia. In our study, 29 patients were children younger than 4 years. Except for three children diagnosed through surgical tumorectomy, all remaining 26 children were diagnosed through a mass spontaneously discharged from the vagina or a biopsy specimen. Biopsy specimens were mostly obtained at the time of hysteroscopy and/or pediatric rhinoscopy. In our hospital, hysteroscopy and pediatric rhinoscopy are used as colposcopy for pediatric patients to inspect the vagina and cervix and to perform tumor site biopsy. In recent years, hysteroscopy has been considered an emerging tool for identifying and treating cervical and vaginal diseases (36). The vaginoscopic approach for hysteroscopy involves introducing a hysteroscope directly into the vagina (37). After distention by a fluid medium, it is possible to carefully inspect the vagina and perform eventual interventions (37). Vaginohysteroscopy and pediatric rhinoscopy, without the aid of a speculum, facilitate detailed endoscopic inspection of the lower genital tract and reduce patient discomfort (38). If the distention of the vaginal cavity is inadequate, it might be useful to manually close the vaginal labia to generate increased pressure and vaginal walls distension (37).

Apart from obtaining a biopsy for diagnosis, vaginohysteroscopy and pediatric rhinoscopy were also used to confirm the efficacy of treatment after normalization of AFP levels. Although serial serum AFP levels were monitored before each cycle of chemotherapy, a final pathologic diagnosis on biopsy is more straightforward to evaluate tumor response. In the present study, 27 patients underwent a second evaluation, mostly consisting of an examination under anesthesia and biopsy of any abnormalities. Repeat biopsy specimens were mostly obtained *via* vaginohysteroscopy and/or pediatric rhinoscopy. Interestingly, yellow, grayish-yellow, or yellowish-white tissue with an irregular shape was observed in 66.7% of the cases during repeated examinations in our study, possibly due to the post-chemotherapeutic presentation of extragonadal YSTs arising in the female genital tract. Although five of the 27 patients still had an elevated serum AFP level before repeat examinations, pathological results revealed no evidence of residual tumor in 25 cases. Therefore, whether to perform further endoscopic assessment after a normalized AFP level remains to be determined (32). Whether AFP can be used as the only trackable indicator for evaluating the therapeutic effect of vaginal YSTs, such as β-human chorionic gonadotropin, in gestational trophoblast diseases, remains to be determined. Repeat examinations with biopsy are certainly warranted in cases in which serum AFP levels decrease at an unsatisfactory rate or any evidence of residual mass is apparent at the end of treatment (31, 32).

Uterine YSTs are unusual compared to vaginal YSTs. To date, few studies on endometrial and cervical YSTs have been reported (7–28). According to previous literature (7–28), the majority of patients with endometrial and cervical YSTs underwent hysterectomy and adnexectomy combined with chemotherapy (mostly a BEP regimen). Some patients with endometrial YSTs undergo additional procedures concurrently, such as omentectomy (8, 12, 17, 21–23), appendicectomy (12, 23), and pelvic and/or para-aortic lymphadenectomy (8, 9, 12, 16, 21–24). However, only a handful of cases of endometrial YSTs involve the ovary (12), omentum (12, 23), appendix (12), and lymph nodes (9). In total, five cases of uterine YSTs were identified in which at least one ovary was preserved (8, 10, 15, 16, 18). Of these five cases, one 30-year-old patient, gravida 2 para 1, retained her right adnexa because no macroscopic abnormality was observed (16), while the other four patients were all nulligravid, and they retained bilateral ovaries (8, 10, 15, 18). Follow-up data from 12 months to 6 years was available for these five patients, and they were all alive and disease-free. One patient underwent oocyte cryopreservation 6 months after completing chemotherapy, retrieving 18 mature oocytes in one cycle (8).

According to limited studies on uterine YSTs, a combination of radical surgery and chemotherapy seems to be the first-line treatment (10, 22, 39) and may result in better survival (22). However, the specific range remains undefined (10, 40) and whether the uterus and ovaries can be preserved still needs further exploration (10, 41). Data on the correlation between radical surgery and survival outcomes are insufficient to define standard guidelines. We believe that preservation of sexual and reproductive function is worth attempting in the management of uterine YSTs, especially in patients with a strong desire for future fertility. The possibility of giving birth and potential risk of delayed treatment should be balanced. Even though patients lose their childbearing potential after hysterectomy, preserving bilateral ovaries when indicated is still a chance to have a genetic child in the future. Moreover, ovary-sparing can retain the patient's ovarian endocrine function and improve postoperative quality of life (10). Finally, careful assessment, informed consent, and strict follow-up should be considered for preservation of the uterus and ovaries.

In our study, all 32 patients underwent combination chemotherapy, and 21 patients were treated with BEP alone. Five patients underwent surgery after chemotherapy, but no evidence of malignancy was found. In total, 31 patients achieved CR after the initial treatment, and two of them experienced recurrence. Three patients died of the disease in periods ranging from 4 to 28 months. One patient died of the disease due to delayed and nonstandard treatments in a local hospital, the second died of a severe adverse effect of chemotherapy, and the third died of recurrent disease. Twenty-nine patients (90.6%) remained disease-free during the long follow-up period. Additionally, among 27 alive patients who retained the uterus and ovaries, one patient recovered her menstruation after completing chemotherapy and five had undergone menarche.

In general, we achieved a favorable prognosis in this cohort. We attributed the excellent prognosis to effective treatments and close cooperation with pediatric doctors and careful pediatric nursing. BEP chemotherapy, administered in time and with sufficient dosage (31, 32), could serve as the sole treatment modality, during which biopsies should be performed to diagnose or evaluate the efficacy of chemotherapy (33). If a suspicious lesion is found during chemotherapy and follow-up, examination under anesthesia with biopsy should be performed. However, caution should be exercised when performing additional procedures. Vaginectomy and THBS can lead to loss of sexual function and fertility. Fibrosis and growth impairment of the vagina secondary to partial vaginectomy have also been reported (42). If any malignancy is suspected, frozen section analysis is helpful to confirm the diagnosis and prevent unnecessary surgery.

Our study has some limitations including bias caused by transferred patients and a large but still limited sample. More than half of the transferred patients initially received non-standard treatment in local hospitals. Two patients underwent surgery prior to chemotherapy. Three patients received nonstandard chemotherapy, and one patient received too many courses of BEP. Moreover, only two uterine YST cases were included in our study, and more cases are needed to explore the feasibility of fertility-sparing treatment for uterine YSTs. Finally, most patients are still premenarchal after long-term follow-up, and a longer follow-up is needed to indicate the outcomes of fertility.

## Conclusion

To preserve sexual function and fertility, BEP chemotherapy can be used for the treatment of vaginal and uterine YSTs with prior consideration. For uterine YSTs, this treatment modality should be chosen with a careful comprehensive assessment and strict follow-up. Vaginohysteroscopy and pediatric rhinoscopy can be used to diagnose and evaluate the chemotherapeutic efficacy in pediatric patients. In most cases, YSTs appear yellow or grayish-yellow after chemotherapy. Biopsies should be performed when indicated during repeat examinations and follow-up. However, any surgery that compromises fertility and sexual function should be performed with caution.

## Data Availability

The original contributions presented in the study are included in the article/Supplementary Material, further inquiries can be directed to the corresponding author/s.
